# Real-Time Performance of a Self-Powered Environmental IoT Sensor Network System

**DOI:** 10.3390/s17020282

**Published:** 2017-02-01

**Authors:** Fan Wu, Christoph Rüdiger, Mehmet Rasit Yuce

**Affiliations:** 1 Department of Electrical and Computer Systems Engineering, Monash University, Wellington Rd., Clayton VIC 3800, Australia; fan.wu@monash.edu; 2 Department of Civil Engineering, Monash University, Wellington Rd., Clayton VIC 3800, Australia; chris.rudiger@monash.edu

**Keywords:** WSN, energy harvesting, supercapacitor, XBee, environmental monitoring, performance

## Abstract

Wireless sensor networks (WSNs) play an increasingly important role in monitoring applications in many areas. With the emergence of the Internet-of-Things (IoT), many more low-power sensors will need to be deployed in various environments to collect and monitor data about environmental factors in real time. Providing power supply to these sensor nodes becomes a critical challenge for realizations of IoT applications as sensor nodes are normally battery-powered and have a limited lifetime. This paper proposes a wireless sensor network that is powered by solar energy harvesting. The sensor network monitors the environmental data with low-power sensor electronics and forms a network using multiple XBee wireless modules. A detailed performance analysis of the network system under solar energy harvesting has been presented. The sensor network system and the proposed energy-harvesting techniques are configured to achieve a continuous energy source for the sensor network. The proposed energy-harvesting system has been successfully designed to enable an energy solution in order to keep sensor nodes active and reliable for a whole day. The paper also outlines some of our experiences in real-time implementation of a sensor network system with energy harvesting.

## 1. Introduction and Related Works

Wireless sensor network (WSN) is increasingly being used to improve applications such as military surveillance, medicine, transportation, environmental conservation, agriculture, home health care, and industrial process control, among others. With the emergence of the Internet-of-Things (IoT), many more low-power sensors will be deployed in different environments to collect, process, analyze, and monitor environmental factors in real time. The power supply of sensor nodes becomes a challenge as sensor nodes are normally battery-powered [1]. Batteries used include primary batteries (non-rechargeable batteries) and secondary batteries (rechargeable batteries). Primary batteries are typically used as the power source for many sensor networks, and the lifetime of these sensor nodes is the time taken to discharge below minimum charge level that a sensor node requires [2]. Although batteries have high energy density, they have a limited lifetime. For long-term deployments, regular battery replacements are required [3]. Battery replacement can be expensive and not feasible in a case like remote environmental monitoring [4,5]. The limited energy also determines some important performance parameters in a sensor network system, such as the sampling frequency and the maximum transmission distance between sensor nodes [6]. With the sampling frequency and transmission power increasing, the sensor node will consume more energy. Three different ways are commonly used in energy-harvesting systems to provide the energy: a supercapacitor, a battery, and a combination of both [7]. To achieve long-term operation, energy harvesting with secondary batteries (or supercapacitors) as an alternative storage element is essential.

Self-powered sensor systems, which are very active research topics, use various energy-harvesting methods, such as solar energy using solar cells [8,9,10] and solar thermoelectric generators [11,12], mechanical energy [13,14], and wind energy [15]. As provided in [16], solar cells can provide the highest power density (15 mW/cm^2^), which is a lot higher than vibration (200 µW/cm^2^), thermoelectric (60 µW/cm^2^), and solar thermoelectric (16 µW/cm^2^) methods [11]. The extraction of solar energy is difficult in nonstationary environments; for example, cloudy days, dust on the solar cell surface, or the varying illumination levels at different times at the day can cause the degradation of performance [17]. Therefore, an energy storage/buffer unit and a solar controller are required. Harvested energy must be stored in an energy buffer. Some works use a supercapacitor as the primary power supply and a battery as the backup source, as studied in [18]. Some others choose rechargeable batteries only. For example, the work in [19] uses one 150 mAh NiMH rechargeable battery as the primary source and a 2200 mAh Li-ion battery as the backup source.

When transferring energy from harvesting sources to the energy buffer, some works use the maximum power point tracking (MPPT) technique [8,20] to keep the solar panel at the maximum power point. Without MPPT, it will become difficult when using a small-sized solar panel to charge the energy buffer. Some commercial chips like LTC3105 [21] and bq25504 [22] can easily support the MPPT function on board.

Supercapacitors have almost unlimited charging cycles, high power density, high charge–discharge efficiency (97%–98%), and they do not release any thermal heat during the discharging process, while rechargeable batteries have lower charging cycles and high energy density [16,18]. These outstanding features make supercapacitors a suitable primary source for sensor nodes in a sensor network system. Research in [23] shows that different supercapacitors have voltage losses from 5%–60% over 2 weeks’ time, and as the temperature increases, the self-discharge rate increases as well. 

A few existing wireless sensor networks use solar energy harvesting. For example, the work in [6] uses a 54 mm × 42 mm solar cell, with a 2.3 V 50 F supercapacitor and rechargeable batteries together as the energy-harvesting unit. The rechargeable battery acts as the backup buffer. With this unit, the power system is enough to extend the operational life of the network to 1 year. They also indicate that having both supercapacitor and battery in the system will increase the feasibility of the network design architectures.

The work in [8] presents an efficient, small-sized, low-cost MPPT circuit for a WSN. The solar energy is transferred from solar panels to batteries, and then the power is supplied to the sensor nodes. This solar-powered WSN monitors the temperature and luminosity information of the marine ecosystem at different depths. A solar-powered power management unit operating with battery packs is implemented to maximize the power efficiency and lifetime. 

Work in [3] presents a WSN for bridge structural health monitoring (SHM). The researchers installed solar panels (11.4 cm × 21.0 cm) and rechargeable batteries with 10,000 mAh capacity in eight nodes, where changing the battery is difficult. They found that for nodes with good sunlight, battery voltage can be well maintained at around 4.15 V to keep the system working; for a node with only indirect or reflected sunlight, the battery voltage keeps decreasing and the solar panel cannot charge the battery.

SensorScope [19,24] presents WSNs for monitoring harsh environments. Solar energy-harvesting has been integrated into each of the stations to power up the sensor nodes and enables the long-term deployment of the system. The system is able to last for 180 days.

Work in [25] also uses solar energy harvesting, whereby it is only used in the gateway node because the gateway consumes more power compared to the sensor node. The gateway is placed inside a birdhouse that is quite useful. This paper also reports that under direct sunlight, it is hard to charge Li-ion rechargeable batteries with a charging temperature ranging from 0–45 °C. The gateway battery voltage keeps decreasing without charging once the open-air nature temperature increases. This is an important issue that needs to be taken into account while selecting the energy storage unit and choosing the casing. In the work in [26], researchers use a solar radiation shield to protect the electronic components from direct sunlight exposure and rain, which may be useful in solving this issue.

Many works utilize solar panels that can generate relatively higher power: for instance, a 1.5 W solar panel is used in work [9] and a 3 W solar panel is used in works [10,27]. The large output-power solar panels can charge rechargeable batteries more easily compared to the solar panel with only a few hundred milliwatts of output power. However, these solar panels normally require a larger area—more than 100 cm^2^—and could be used together with the bigger-sized sensor nodes.

The work in [10] deploys a solar-powered traffic-sensing network in urban and desert areas, which uses an XBee module as the communication module. As described before, the sensor node is powered by a 3 W solar panel, which is far enough for powering a 90 mW sensor node. However, the author finds that in practice, the sensor node ends up running out of energy quite fast due to dust accumulation on the solar cell and shadows from surrounding buildings. As the temperature gets high, the Li-ion batteries used in the node are also affected severely. 

The systems mentioned above use large solar panels and do not study the performance of the sensor network with solar energy harvesting in detail. We have designed a solar energy-harvesting unit and integrated it with EN-Nets (a low-power environmental sensor network system developed at Monash University, Australia). The proposed energy-harvesting mechanism can successfully supply the required power to sensor nodes in real time, extending the lifetime of the WSN. Supercapacitors or rechargeable batteries are used as the storage unit to hold the required amount of harvested energy for continuous operation. The proposed technique provides an energy solution to keep sensor nodes active for a whole day. Each sensor node includes multiple sensors, one microcontroller, one XBee module, and one energy-harvesting unit. The system has been validated in both field and laboratory environmental conditions.

The remainder of this paper is organized as follows: Section 2 outlines hardware implementation; Section 3 discusses some results and evaluations, and a brief conclusion and possible future improvements are given in Section 4.

## 2. Hardware Implementation

**Figure 1:**
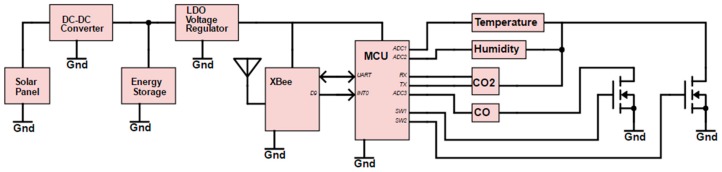
Schematic diagram of the self-powered environmental monitoring sensor node.

**Figure 2:**
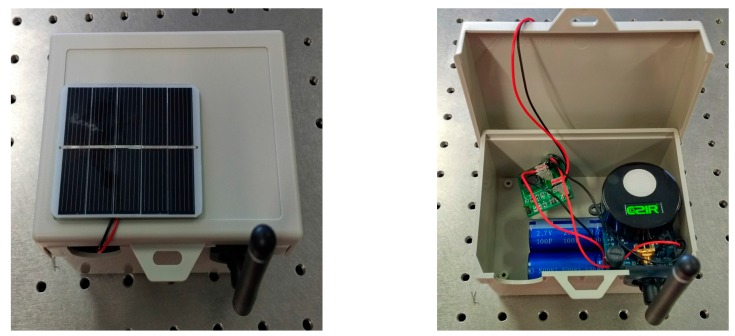
Sensor nodes with solar energy harvesting.

A simplified schematic diagram of the self-powered sensor node is shown in Figure 1. The energy-harvesting system sources power from a small-sized solar panel and then provides the power to the power management unit in a sensor node. Power management includes one low drop-out (LDO, MCP1700 from Microchip Technology Inc., Chandler, AZ, USA) voltage regulator to regulate the sensor node voltage, and one microcontroller with three MOSFET (metal-oxide semiconductor field-effect transistor) switches to turn the different sensors on and off for a specific time to reduce power consumption (only two switches are shown in the figure because only two switches were used in this experiment). The DC–DC converter helps to keep the voltage constant and charges rechargeable batteries or supercapacitors. The wireless sensor node operates with a small duty cycle, D = 1.25% (a 15 s wake-up time in a 20 min period). Therefore, the aim of the energy-harvesting system is to provide sufficient power to run for at least 1 day once the supercapacitor has been charged to a certain capacity. The size of the harvesting prototype board is 3 cm × 3 cm and it can be placed inside the sensor box together with the sensor node and supercapacitors. A detailed description is provided below. Figure 2 shows the sensor node with the solar panel.

### 2.1. Energy Harvesting Unit

**Figure 3:**
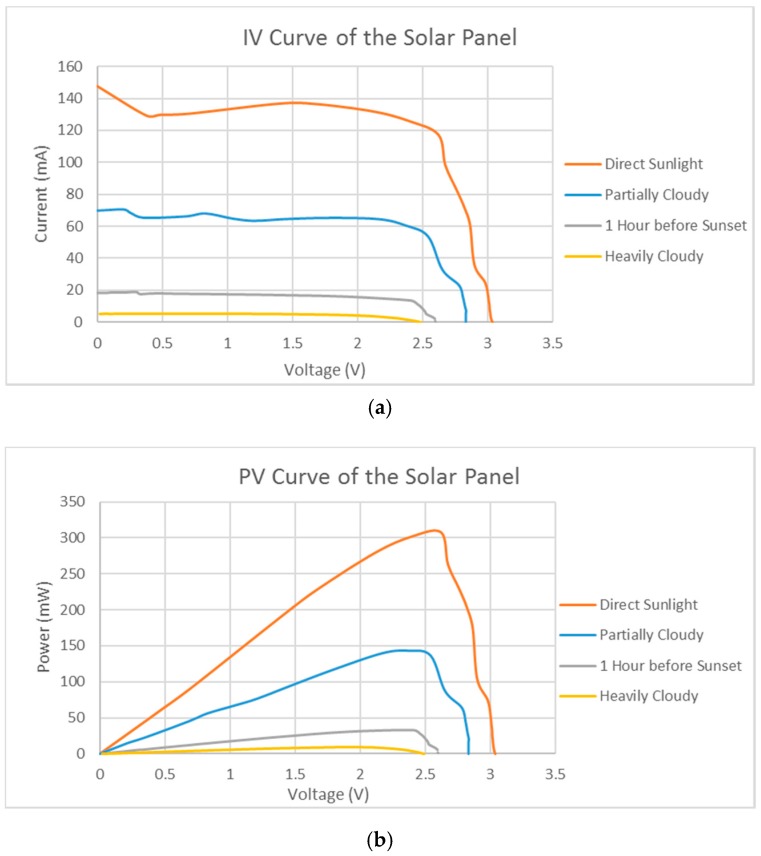
(**a**) Current–voltage (IV) curve of the solar panel; (**b**) power–voltage (PV) curve of the solar panel.

A 55.0 mm × 67.5 mm × 3.2 mm solar panel (from Shenzhen Chuanningsheng Electronics Co., Ltd., Shenzhen, China) is used in the system. The solar panel features open voltage at 3 V and short-circuit current at 150 mA. The maximum power it can produce is around 310 mW with a voltage of 2.6 V. The characteristic diagrams are measured under direct sunlight, on a cloudy day, 1 h before sunset, or under heavily cloudy conditions using different load resistors, which are shown in Figure 3. The current is measured by connecting a current-meter in a series with the load resistor. The voltage is measured as the voltage difference across the resistor.

LTC3105 is used in the circuit to track the maximum power point of the solar panel. This device contains a high-efficiency step-up DC–DC converter with a low start-up voltage (250 mV) that can support small-sized solar panels and features a maximum power point controller (MPPC) function. The maximum power point (MPP) is set by changing the resistance of the MPPC resistor to suit the solar panels’ MPP, which is around 2.5 V. The output voltage is also user selectable. In our design, the output is set at 4.65 V when charging the supercapacitor and at 4.1 V when charging the rechargeable battery.

Setting MPP makes the solar panel only work at maximum power point 2.5 V. Therefore, the solar panel works at around 300 mW under direct sunlight, 150 mW on a partially cloudy day, and around 23 mW 1 h before the sunset. During a heavily cloudy day, the output power of the solar panel is near 0 at 2.5 V.

Initially, our sensor nodes were designed to be powered by batteries. Therefore, a LDO voltage regulator was added to the sensor node to regulate the input voltage. It requires at least 3.6 V in order to keep the sensor node running at a nominal voltage of 3.3 V.

For supercapacitors, the energy is stored electrostatically and does not involve any chemical reactions. One drawback of a supercapacitor is that it has a higher self-discharge current, which is 0.28 mA for the one used in the work (HP-2R7-J107UY LR from Jinzhou Kaimei Power Co. Ltd. [28]). While supercapacitors are normally restricted to low voltages ranging from 2.5 V to 2.7 V for a higher capacity, they must be connected in series to get higher voltages. We used two 100 F × 2.7 V supercapacitors in series to obtain 50 F capacitance with a maximum voltage of 5.4 V. For rechargeable batteries, 18650 rechargeable batteries (ICR18650-26F from Samsung SDI Co., Ltd., Yongin, South Korea) were chosen, which each have 2600 mAh capacity.

### 2.2. Brief Descriptions of Sensor Node

The main components of the sensor node include one XBee module, one microcontroller unit (MCU), and four sensors on the sensor board in this testing. XBee-Pro 900HP (from Digi International Inc, Minnetonka, MN, USA) is a low-power module that consumes only 2.5 µA during sleep time. The MCU in this work is ATmega328p (from Atmel), which features low power, low cost, and high performance. 

The four sensors were carbon dioxide (CO_2_), carbon monoxide (CO), temperature, and humidity. The temperature and humidity sensor were both soldered on the board, while CO_2_ and CO sensors were removable. The temperature sensor (MCP9700 from Microchip) is an analog sensor that can be connected to the analog-to-digital conversion (ADC) pin of the MCU. It can be powered from 2.3–5.5 V with only 0.6 µA current consumption. The humidity sensor (HIH5030 from Honeywell International Inc, Morris Plains, NJ, USA) is an analog low-voltage sensor, which operates from 2.7 V to 5.5 V with 200 µA current consumption. The CO sensor (MICS-5121WP from SGX Sensortech Limited) is also an analog output sensor, which has a wide detection range from 1 to 1000 ppm. It is the most power-hungry sensor in our design, which consumes 30.7 mA current. The CO_2_ sensor (GC-0012 from CO_2_ Meter) is an ultralow power consumption (3.5 mW) sensor which can measure 0–10,000 ppm CO_2_ concentration. This sensor supports digital output and is read by the MCU. Temperature, humidity, and CO_2_ sensors were controlled by the SW2 and woke up for 15 s, while the CO sensor was controlled by the SW1 and was turned off immediately after the reading had been taken to save power.

## 3. Results and Evaluations

### 3.1. Power Requirement of Sensor Nodes

Table 1 represents the current requirements for each component of the sensor node. The design of the solar-harvesting board should be able to ensure the supply of sufficient energy for them. 

**Figure 4:**
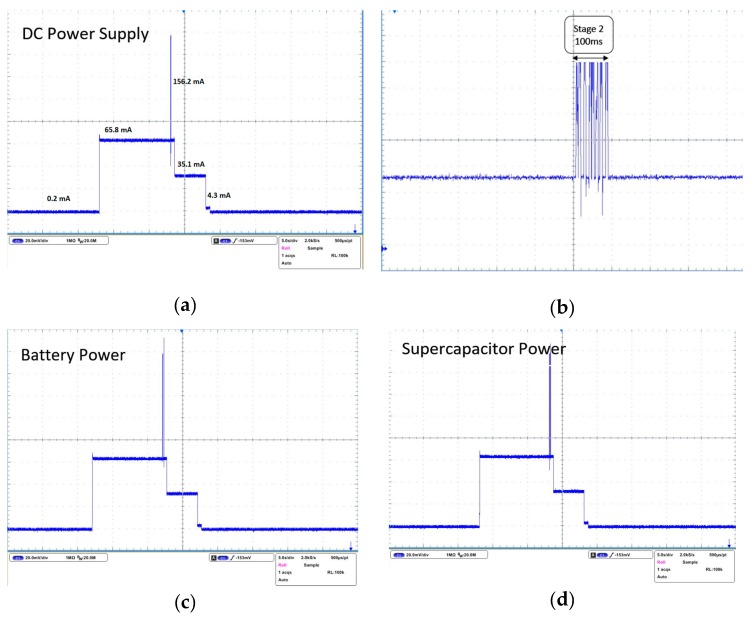
(**a**) Current waveform from DC power supply; (**b**) zoom-in of stage 2; (**c**) current waveform from battery power; (**d**) current waveform from supercapacitor.

The sensor node was set to have 19 min and 45 s of sleep time and 15 s of transmission time. To obtain the current waveform, a 1 Ω resistor was connected between the energy-harvesting board and the sensor node. Then, the oscilloscope was used to obtain the voltage difference across the resistor, and the current was then measured by dividing V by 1. Figure 4 shows the current waveform of a sensor node when it is operating and supplied by different power supply methods. Since the maximum current of the sensor node was 156.2 mA, the voltage drop across the resistor was only 0.1562 V. This did not affect the sensor node’s performance since we were trying to get the current waveform only. The sensor node’s wake-up time includes stage 1, stage 2, and stage 3, each lasting for 15 s in total. The sensor node’s sleep time includes stage 4 and stage 5, adding up to 19 min and 45 s. 

At stage 1, the sensor node was in idle mode with all the sensors running, and it drew a 65.8 mA current, which consumed 217.14 mW power. At stage 2, the XBee was in transmit mode for around 100 ms (as shown in the zoomed-in graph) and the sensor node drew 156.2 mA with 515.46 mW power consumption. At stage 3, the CO sensor was turned off by the MCU at 10.5 s after the reading was taken by XBee device in order to save more power and make sure that the CO value had been recorded. As a result, the current consumed by a sensor node dropped from 63.7 mA to 33 mA. Stage 4 represents that the XBee module went into sleep mode, whereas the MCU was still awake for another 1 s, which draws 4.3 mA current only. At stage 5, the MCU went into sleep mode as well, and the sensor node only drew 0.2 mA. This stage lasted until the next wake-up cycle of the sensor node. This is extremely low power, considering the long-term monitoring of power-hungry sensors such as CO sensor. All the currents and power consumptions have been tabulated in Table 2. 

The power supply unit plays a critical role in wireless sensor networks. We tested whether different types of power supply units will have different effects on the sensor nodes. In Figure 4a,c,d, the power sources are DC power supply, battery, and supercapacitors, respectively. Three types of power supply do not have any effects on the sensor nodes. It can be concluded that the nodes directly powered from large supercapacitors demonstrate the same current waveform and performance.

### 3.2. Power Calculations and Capacitor Selection

In theory, the total power consumption of the entire sensor node can be calculated using Equation (1). The sensor node’s power consumption at each stage can be denoted by P_1_, P_2_, P_3_, P_4_, and P_5_. P_supercap_ is the power loss from the self-discharge of the supercapacitor. P_vrl_ is the power dissipation resulting from the voltage regulator loss.

(1) $$P=P_{1}+P_{2}+P_{3}+P_{4}+P_{5}+P_{supercap}+P_{vrl}$$

The sampling frequency for the sensor node was 20 min. Each sensor node would wake up for 15 s every 20 min. Here, a time period of 20 min (1200 s) is used to calculate the power consumption. The total average power consumption of the sensor board can be calculated as follows:

(2) $$\begin{array}{l} P_{1}+P_{2}+P_{3}+P_{4}+P_{5}\\ =\frac{217.14mW\times 10.4s+515.46mW\times 0.1s+115.83mW\times 4.5s+14.19mW\times 1s+0.66mW\times 1184s}{1200s}\\ \approx 3.022\;mW \end{array}$$

During the discharging time, the voltage of the supercapacitor was linearly decreasing from 4.6 V to 3.6 V. Therefore, we assume that the average voltage is equal to ½ (4.6 + 3.6) = 4.1 V. The supercapacitor’s self-discharge power consumption can be calculated as follows:

(3) $$P_{supercap}=\frac{4.1V\times 0.28mA\times 1200s}{1200s}=1.148\;mW$$

The power dissipation between the voltage regulator’s input and output was also linearly decreasing from 1.3 V to 0.3 V as the supercapacitor voltage decreased from 4.6 to 3.6 V. The average voltage change can be calculated as ½ (1.3 + 0.3) = 0.8 V. The average current for the sensor is equal to the total power of the sensor board divided by 3.3 V, which is equal to 0.921 mA. Therefore, the power dissipation loss from the voltage regulator is calculated as follows:

(4) $$P_{vrl}=\frac{0.8V\times 0.921mA\times 1200s}{1200s}=0.737\;mW$$

The total average power consumption was equal to 3.022 + 1.148 + 0.737 = 4.907 mW. This average power consumption can be lowered by reducing the duty cycle or increased by increasing the duty cycle.

The average annual daylight duration is 12 h. Therefore, to achieve 12 h of continuous power supply by the supercapacitor while it is dark, the required capacitance can be calculated as follows:

(5) $$C=\;∆t\times 2\times \frac{P}{(Vi^{2}}-Vt^{2}\;=12\times 3600\times 2\times \frac{4.907}{4.6^{2}-3.6^{2}}\;\approx 51.7\;F\;$$

Therefore, in theory, the capacitor with 51.7 F capacitance can support the sensor node running for 12 h without any problem once it is fully charged. The nearest whole number to 51.7 F of supercapacitance is 50 F. Therefore, we used a 50 F capacitor in this design. There is a trade-off between the size of the solar panel and the energy storage capacity [4,5]. During the winter, when the average daylight hours in Melbourne are shorter than 12 h, a higher capacitance value would be required.

### 3.3. Supercapacitor Charging and Discharging

The charging rate for the 50 F capacitors is shown in Table 3. It was charged from 0 V onwards. The voltage increased 2.17 mV per second. It took around 45 min to charge from around 0 V to 4.6 V while there was direct sunlight on the solar panel.

**Figure 5:**
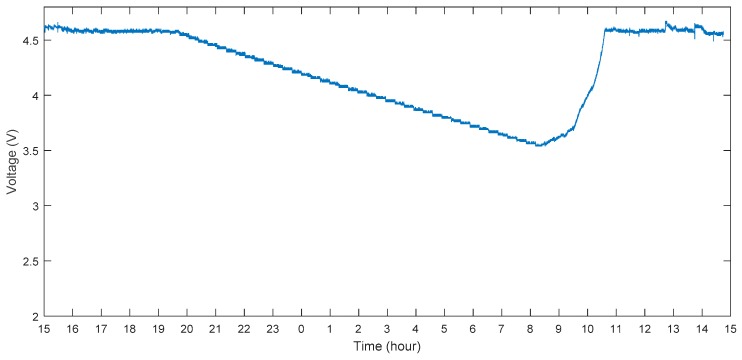
Continuous supercapacitor voltage-monitoring from 15:00 to 15:00 AEDT (Date: 18 October 2016, Clayton Victoria, Australia).

Figure 5 shows the continuously monitored supercapacitor voltage changing. The experiments were conducted in Clayton, Victoria (37.91° S and 145.13° E). We used one Arduino Uno to monitor the voltage changing every second and plotted the graph. The supercapacitor was fully charged at 4.6 V before the deployment. The voltage could be maintained at 4.6 V from 15:00 to 19:45 because there was still sunlight during this time. After 19:30, the voltage decreased linearly and this trend lasted for approximately 12 h, which is similar to the calculation in Section 3.3. During the early morning and with enough solar insolation, the supercapacitor could charge slowly from 3.6 V to 4.6 V within 2 h. When it was fully charged and had enough solar insolation, the voltage stayed almost constant and without dropping.

### 3.4. Integration of Solar Energy Harvesting and Sensor Node

**Figure 6:**
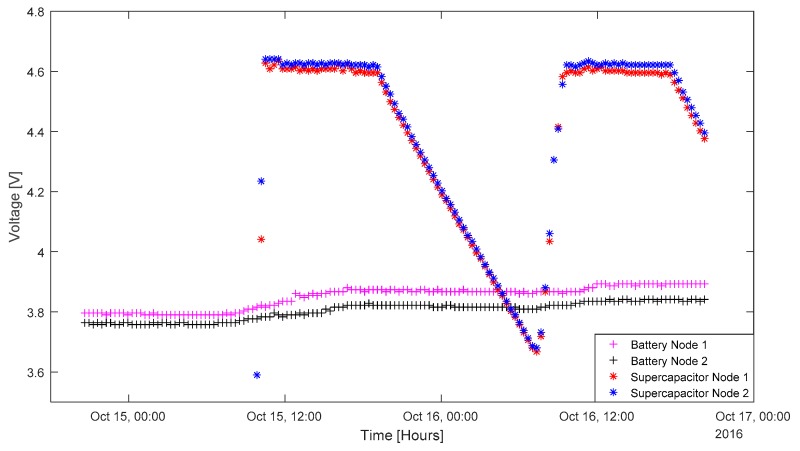
Energy storage unit voltage monitoring.

To observe how voltage changes, two sensor nodes powered by batteries and two sensor nodes powered by supercapacitors (solar-powered) are used. The rechargeable batteries are without charge before and the supercapacitors were discharged before the testing. Figure 6 shows the differences between the battery voltage and the supercapacitor voltage change for 2 days. All the voltage readings were monitored by the sensor node and sent to the base station via a radiofrequency (RF) packet. This experiment started at around 8:20 p.m., when there was no sunlight. All the sensor nodes were deployed at the same time. As for the two battery-powered sensor nodes, they started to send data to the base station after 20 min of deployment, which was coordinated by the network coordinator. This was because their initial voltage was just under 3.8 V, and this was enough for powering up the sensor node. The two supercapacitor nodes started to send data to the base station at around 10:00 a.m. in the morning of the second day. This was because, before the deployment, the supercapacitors were discharged to under 1 V. It took some time for the energy-harvesting board to charge them back to 3.6 V in the morning. It took only 20 min for the supercapacitor Node 1 to charge from 3.6 V to 4.235 V. In the next wake-up time, the voltage had already been charged to above 4.6 V. This was similar for the supercapacitor Node 2; within 20 min, the voltage had already been charged from 4.0 V to 4.6 V. 

Overall, we can also see that the battery voltage gradually increased in the daytime and remained almost constant in the night. The supercapacitor voltage could be kept constant once it is fully charged in the morning, and decreased linearly at night. The node powered by the supercapacitor could join the network by itself at a later stage, even if it was not synchronizing with the network at the start of the deployment. Both the supercapacitor and the battery were enough to power the sensor node. Once it had been fully charged, the 50 F supercapacitor could continuously supply power to the sensor node during nighttime. 

### 3.5. Sensor Network Performance

With the solar energy-harvesting system, when the sensor nodes were placed under sunlight, it resulted in some issues for temperature readings and humidity readings of the sensor nodes. Ten sensor nodes were placed in similar locations, where they could receive direct sunlight in the day. For all battery-powered sensor nodes, batteries had initial voltage above 3.7 V. For the supercapacitor-powered nodes, Nodes 1 and 3 had an initial voltage above 4 V; Nodes 2, 4, and 5 were discharged before the deployment.

**Figure 7:**
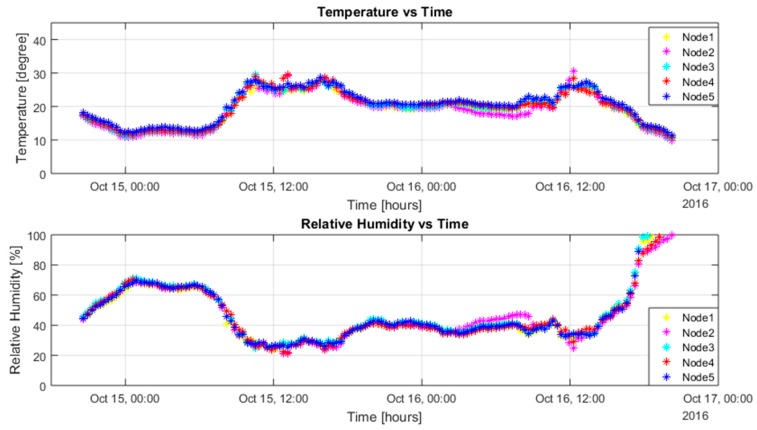
Battery-powered sensor nodes’ performance.

**Figure 8:**
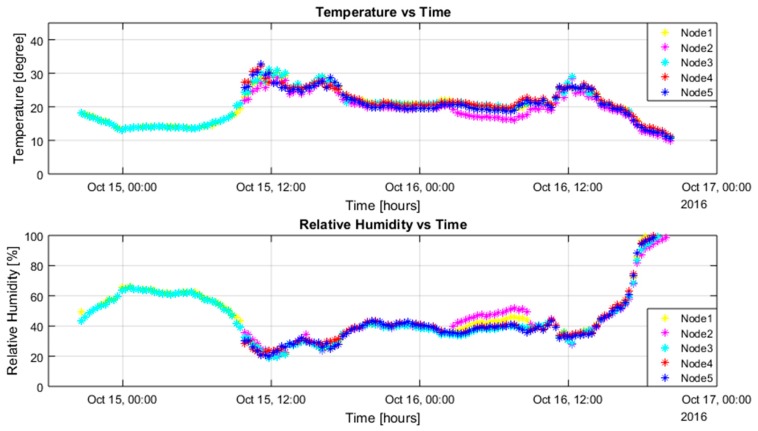
Supercapacitor-powered sensor nodes’ performance.

Overall, the readings are quite similar and match each other. Under sunlight, the internal temperature of the sensor box increased a lot, which caused the temperature sensor reading to increase. From Figure 7 and Figure 8 above, we can see that the temperature reading could increase above 30 °C. The maximum temperature on the day of testing was about 27 °C. The humidity value was calibrated by the temperature value, which made it slightly inaccurate as well. At the end of testing, the temperature value decreased to below 10 °C and the relative humidity values increased significantly to 100%. This was because during that time, it was raining in Melbourne, which caused the relative humidity to rise.

For the networking performance, for all battery-powered sensor nodes, all the RF packets were received successfully, which were 145 packets from each sensor node in total. For the supercapacitor-powered sensor nodes, Nodes 1 and 3 successfully sent out 145 packets from each of them, which were the same as battery nodes; Nodes 2, 4, and 5 sent out 105 packets from each node after they had been charged during the morning of the second day. The network reliability under the energy-harvesting system was almost 100% without any RF packet being dropped. 

## 4. Conclusions and Possible Future Improvements

An autonomous IoT network system using solar energy harvesting has been presented in this work. The proposed technique provides an energy solution to keep sensor nodes active and reliable for a whole day. 

For supercapacitor-powered nodes, during the day with sunny or partially cloudy weather conditions, the sensor node could run at least 8 h without interruption. At night, the 50 F supercapacitor could support the sensor node for the whole night until dawn the next day. On the second day of testing, the weather was partially cloudy and the voltage could still be maintained at 4.6 V in the day. However, if there is going to be heavy cloud coverage for a several days, the 50 F supercapacitor-powered node may be offline because the power extracted from the solar panel is close to 0 in this weather condition. To avoid the sensor node being offline, a much higher supercapacitor value is needed in order to provide the required energy to the node during such days. When the supercapacitor is fully charged to the programmed level, the energy-harvesting board almost stops sourcing energy from the solar panel. Some future improvements to utilize the solar energy better, in case of excessive energy, could involve increasing the sampling frequency and transmission power in order to have a more continuous monitoring sensor network and improve the network coverage area. The energy stored in the supercapacitor (E) was equal to ½ CV^2^. Therefore, when the supercapacitor was disconnected at 3.6 V, there still remained ½ × 50 × 3.6^2^ = 324 J of unused energy. A practical solution to maximize the utilization of the energy would be integrating a buck-boost voltage regulator.

For battery-powered nodes, a small-sized solar panel could successfully charge the large-capacity rechargeable battery, which was 2600 mAh. The battery voltage could gradually increase from the time we deployed the sensor nodes and could be maintained at a certain level during the night. This also fulfilled our expectations of charging the battery and keeping the sensor nodes running without disruption.

Battery-powered nodes can start to communicate with the base station right after the deployment because a new rechargeable battery has an initial voltage about 3.8 V Nevertheless, for supercapacitor-powered nodes, there is a delay in network connection if the supercapacitor is deployed at night. For the network that we set up in this experiment, it took approximately 11.5 h (8:20 p.m. to 10:00 a.m.) to join and synchronize with the whole network. To enhance the performance of supercapacitor-powered nodes, all the supercapacitors can be charged before the deployment. 

The maximum power that can be extracted from the solar panel is 300 mW (sunny day), 150 mW (partially cloudy day), and 23 mW (1 h before sunset). The sensor node only draws an average power of 4.9 mW. In the worst month (June), the daily peak sunlight hours are 2.4 h in Melbourne [29]. Considering that the harvesting board is running at 300 mW for 2.4 h only, the average power from the solar panel will be (300 × 2.4)/24 = 30 mW. Although 30 mW is still oversized for sensor nodes, the sensor node can utilize more solar power if needed when the duty cycle of the network is increased.

A better solution for the WSN would be having two separate energy supply units. One would have a supercapacitor as the main source and a rechargeable battery as the backup source. Whenever the supercapacitor’s voltage drops below a certain level, the control circuit will switch the power supply from the supercapacitor to the battery so that the network will keep on running and prolong the lifetime of the sensor node. Our future goals for the WSN will be improving the energy-harvesting unit so that it can have a more robust control circuit to manage the power supply unit and improving the sensor box so that direct sunlight will not cause the internal temperature to increase too much.

## Figures and Tables

**Table 1:** Power consumption for each component on the sensor node (current value from datasheet).

Function	Device	Wake-Up Time (s)	Idle/transmit Current (mA)	Sleep Current (µA)
RF module	XBee	15	29/120	2.5
MCU	ATMega328	15.5	4.35	200
Humidity	HIH5030	15	0.2	0
Temperature	MCP9700	15	0.006	0
CO_2_	GC-0012	15	1.5	0
CO	MiCS-5121WP	10.5	30.7	0
LDO	MCP1700	15.5	0.0016	1.6
	Total		65.7576/156.7576	204.1

RF: radiofrequency; MCU: microcontroller unit; LDO: low drop-out.

**Table 2:** Power consumption for sensor node device (actual measurements using multimeter).

Stage	Mode	Current (mA)	Power Consumption (mW)	Energy Consumption (mJ)
1	Idle (0–10 s, 10.1–10.5 s)	65.8	217.14	2258.26
2	Transmit (10–10.1 s)	156.2	515.46	51.55
3	Idle (10.5–15 s)	35.1	115.83	521.24
4	Sleep (15–16 s)	4.3	14.19	14.19
5	Sleep (16 s+)	0.2	0.66	781.44

**Table 3:** Charging rate for the 50 F supercapacitor.

Time Intervals (min)	Charging Rate (mV/s)	Voltage (V)
0–10	2.17	0–1.3
10–20	2.00	1.3–2.5
20–30	1.67	2.5–3.5
30–45	1.22	3.5–4.6
